# Novel Peptide CM 7 Targeted c-Met with Antitumor Activity

**DOI:** 10.3390/molecules25030451

**Published:** 2020-01-21

**Authors:** Chunlei Xia, Ying Wang, Chen Liu, Liwen Wang, Xinmei Gao, Dongping Li, Weiyan Qi, Roujin An, Hanmei Xu

**Affiliations:** 1 The Engineering Research Center of Synthetic Polypeptide Drug Discovery and Evaluation, Jiangsu Province, China Pharmaceutical University, Nanjing 211198, China; chunlxia@126.com (C.X.); wy_cpu@126.com (Y.W.); hsdalan@163.com (C.L.); lww_912@163.com (L.W.); 15189806987@163.com (X.G.); ldp147@126.com (D.L.); qiwy05@126.com (W.Q.); autumn0266@126.com (R.A.); 2 Department of Marine Pharmacy, China Pharmaceutical University, Nanjing 211198, China; 3 State Key Laboratory of Natural Medicines, Ministry of Education, China Pharmaceutical University, Nanjing 211198, China

**Keywords:** peptide, antitumor drug, c-Met, receptor tyrosine kinase, targeted therapy

## Abstract

Anomalous changes of the cell mesenchymal–epithelial transition factor (c-Met) receptor tyrosine kinase signaling pathway play an important role in the occurrence and development of human cancers, including gastric cancer. In this study, we designed and synthesized a novel peptide (CM 7) targeting the tyrosine kinase receptor c-Met, that can inhibit c-Met-mediated signaling in MKN-45 and U87 cells. Its affinity to human c-Met protein or c-Met-positive cells was determined, which showed specific binding to c-Met with high affinity. Its biological activities against MKN-45 c-Met-positive cells were evaluated *in vitro* and *in vivo*. As a result, peptide CM 7 exhibited moderate regulation of c-Met-mediated cell proliferation, migration, invasion, and scattering. The inhibitory effect of peptide CM 7 on tumor growth *in vivo* was investigated by establishing a xenograft mouse model using MKN-45 cells, and the growth inhibition rate of tumor masses for peptide CM 7 was 62%. Based on our data, CM 7 could be a promising therapeutic peptide for c-Met-dependent cancer patients.

## 1. Introduction

The cell mesenchymal–epithelial transition factor (c-Met), also referred to as MET or hepatocyte growth factor (HGF) receptor, belongs to a subfamily of receptor tyrosine kinases (RTKs) and is a transmembrane protein encoded by the *MET* proto-oncogene [1]. It is a heterodimer linked by a disulfide bond between a 50 kDa α subunit and a 145 kDa β subunit [2,3]. The extracellular domain of mature c-Met protein is mainly composed of three functional domains, namely, a semaphorin (Sema) domain (25–516), a plexin–semaphorin–integrin (PSI) domain (517–562), and four immunoglobulin-like regions in the plexins and transcription factors (IPT) domain (563–932). In the extracellular region of the c-Met protein, the PSI domain is connected by four IPT domains to the transmembrane helix of MET and the intracellular kinase domain. The intracellular region of c-Met includes three portions: a juxtamembrane sequence, a catalytic region, and a carboxy-terminal multifunctional docking site. It has been reported that HGF mainly binds to the MET Sema domain, thus inducing the activation of c-Met kinase [4]. At present, the specific role of the IPT domain in the c-Met signaling pathway is still unclear, but it has been reported that the IPT domain of c-Met plays an important role in the activation of c-Met kinase, especially the fourth IPT domain [5,6]. 

HGF, known as a scatter factor, is reported to be the only known high-affinity natural ligand for c-Met. Existing studies have found that activation of the c-Met receptor tyrosine kinase signaling pathway mainly has two forms. One is the HGF-dependent mechanism, namely, HGF autocrine stimulation; the other is the HGF-independent mechanism, such as *MET* gene amplification, receptor overexpression, or *MET* gene fusion. HGF is a pleiotropic cytokine, and mature HGF is a heterodimer composed of an α/β chain, and they are all required for biological functioning [6]. Its β chain has a low affinity with the c-Met binding site in the Sema domain, and the α chain has a high affinity with c-Met, but the exact binding site of the α chain and c-Met is not yet clear. In addition, it was found that the structural domains of c-Met IPT 3 and 4 play a crucial role in the activation of c-Met signaling [6]. 

Precision medicine and target-based therapies have dramatically changed cancer treatment over the past decade [7]. Numerous targeted agents that are already in clinical trials or approved for marketing are designed to block relevant signaling pathways that are important for tumorigenesis, progression, and angiogenesis [8]. Among many targets, the c-Met receptor tyrosine kinase and its ligand hepatocyte growth factor have attracted much attention. The c-Met signaling pathway has been reported to be inappropriately activated in many human solid malignancies and to regulate tumor formation, survival, proliferation, motility, and morphogenesis, which correlate with poor prognosis and even affect tumor metastasis and resistance to target therapy [9,10,11]. Previous studies have reported that the c-Met signaling pathway plays a crucial role in embryo development and tissue regeneration, which is the basis of wound repair, cell morphogenesis, and tumor metastasis [12,13,14]. The proportion of *MET* gene amplification or protein overexpression in Chinese patients with gastric cancer is about 6% and 13%, respectively [15]. Both HGF and its receptor, the tyrosine kinase c-Met, have proved to be a promising target for cancer therapy or diagnosis [16,17,18], but their interactions are complex and remain poorly understood, so they need to be further explored and studied. Currently, drugs targeting c-Met are mainly small-molecule drugs, which are highly toxic and have considerable side effects and production costs. Compared with small-molecule drugs, peptide drugs are safer and less toxic. In addition, it has been reported that the peptide C7 has a good inhibitory effect on hepatocellular carcinoma metastasis [19]. Therefore, the development of peptide drugs targeting c-Met has important research significance and application value. 

In our study, we designed a series of novel sequences of peptides and selected one of them with relatively high affinity to c-Met by computer simulation for further research. Then, the peptide CM 7 was synthesized to evaluate the antitumor activity *in vitro* and *in vivo*.

## 2. Results

### 2.1. Design and Virtual Screening of c-Met-Targeting Peptides and Their Interactions with c-Met

**Figure 1:**
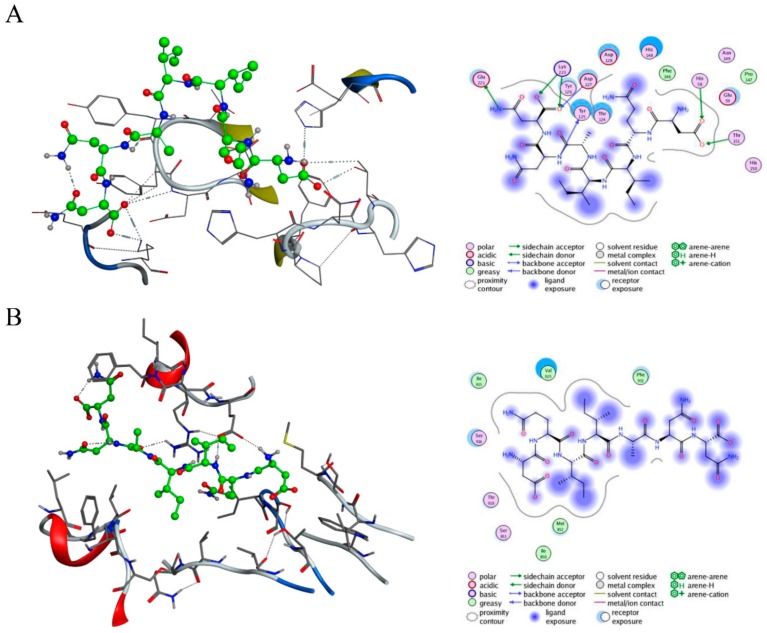
Combination diagram of CM 7 and c-Met. CM 7 is shown in green and c-Met is in gray. (**A**) The interaction between CM 7 and the Sema domain of c-Met (left), and the action site map of CM 7 and the Sema domain of c-Met (right). (**B**) The interaction between CM 7 and the IPT domain of c-Met (left), and the action site map of CM 7 and the IPT domain of c-Met (right).

Relevant studies have found that abnormal activation of the c-Met signaling pathway is induced and regulated by HGF, and studies have also reported that the immunoglobulin-like region, especially IPT 3 and 4, or the Sema domain of c-Met plays a crucial role in the aberrant activation of this signaling pathway [6]. Therefore, we mainly designed peptides by taking the HGF/c-Met binding site and the Sema or IPT domains (IPT 3 and 4) as binding sites. We designed a virtual peptide library based on the structure and interactions between HGF and c-Met, the Molecular Operating Environment (MOE) software (Chemical Computing Group, Quebec, Canada) was used to analyze the interaction between different molecules, and the established analogue database was subjected to virtual screening, as described in the Methods section. The results showed that the affinity between c-Met and CM 7 (DQIIANN) was highest (Table 1), and the interaction between CM 7 and c-Met is shown in Figure 1. Therefore, CM 7 was selected and synthesized for further study. Further, CM 14 was synthesized as a negative control for special binding experiments.

### 2.2. Synthesis and Detection of the c-Met-Targeting Peptide CM 7 and the Random Sequence CM 14

**Figure 2:**
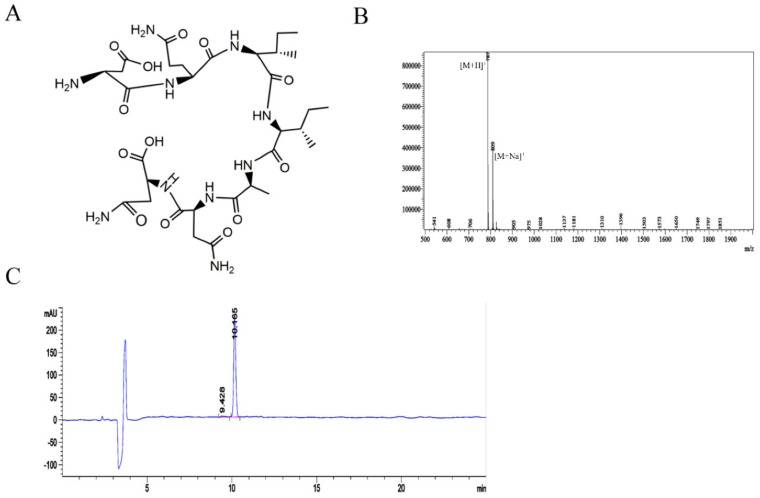
The chemical structure, molecular mass, and purity of peptide CM 7. (**A**) Chemical structure of peptide CM 7. (**B**) Electrospray ionization mass spectrometry (ESI-MS) showed that the molecular mass of peptide CM 7 was 786. (**C**) The purity of peptide CM 7 was determined by high-performance liquid chromatography (HPLC) to be 97.4%.

All the peptides were synthesized by solid-phase synthesis. The selected peptide CM 7, as mentioned above, was synthesized by solid-phase synthesis (Figure 2A). High-performance liquid chromatography (HPLC) was used to detect the purity of the peptide CM 7, and the molecular mass of the peptide CM 7 was determined by mass spectrometry. As shown in Figure 2B,C, the electrospray ionization mass spectrometry (ESI-MS) analysis results showed that the molecular mass of peptide CM 7 was 786, which was consistent with the expected value, and the purity of peptide CM 7 that we synthesized was 97.4%. Fluorescein isothiocyanate isomer (FITC)-CM 7 and FITC-CM 14 were the peptides that were conjugated with FITC at the N-terminus, and the structure, molecular mass, and purity are shown in Figures S1 and S2.

### 2.3. Affinity of the c-Met-Targeting Peptide CM 7 or CM 14 with Its Target 

**Figure 3:**
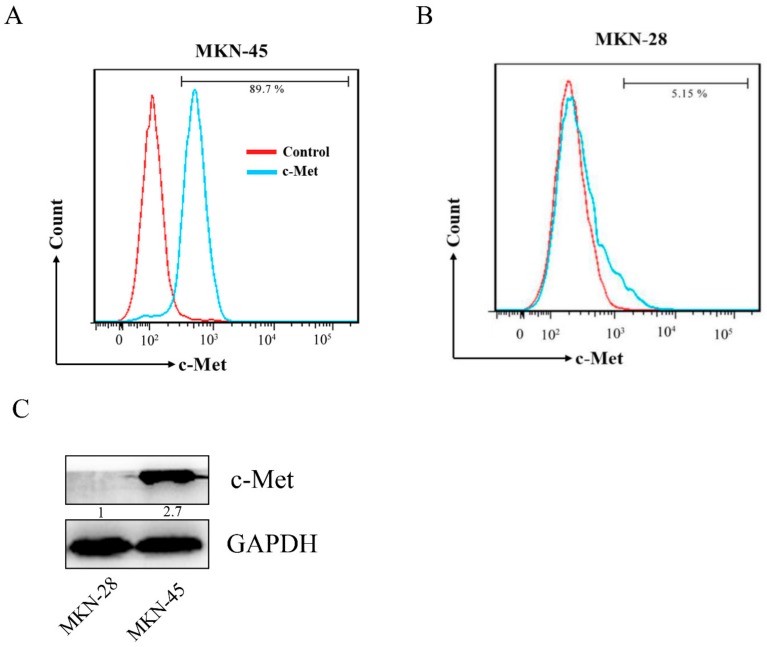
The expression of c-Met in MKN-45 and MKN-28 cells was determined by flow cytometry (**A,B**). (**C**) The expression of c-Met in MKN-45 and MKN-28 cells was determined by Western blot.

**Figure 4:**
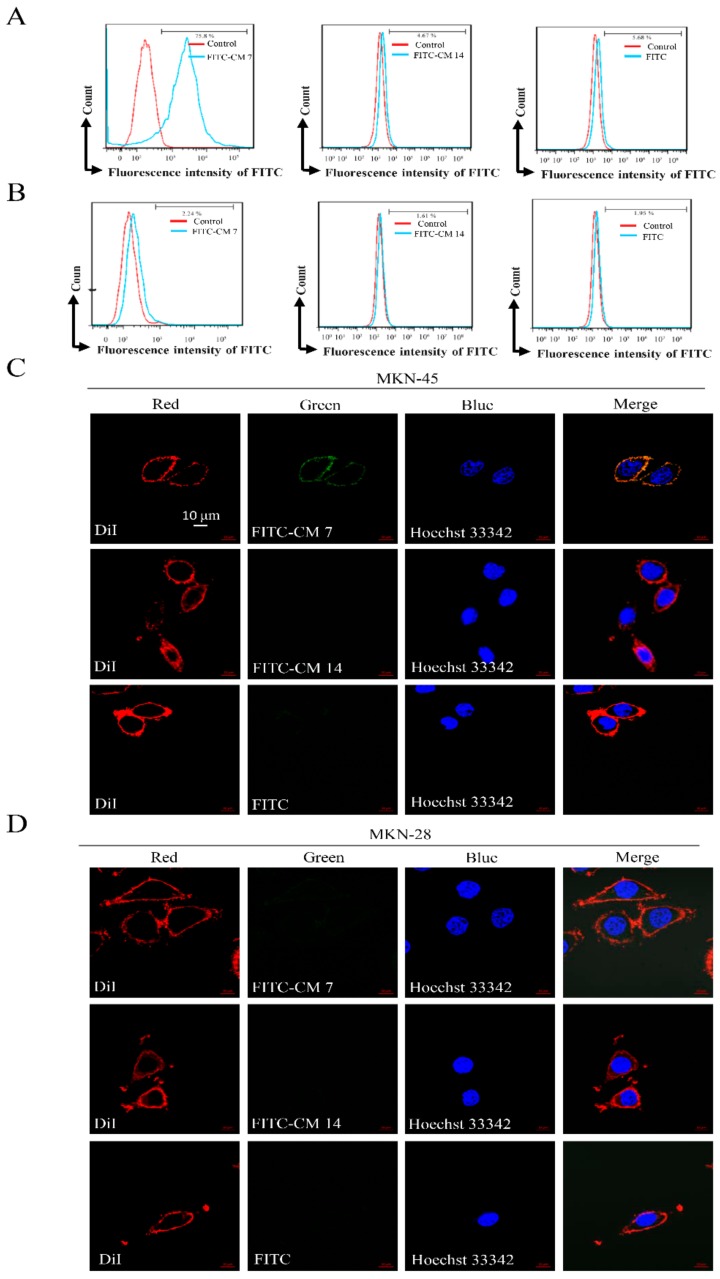
CM 7 binds preferentially to c-Met-enriched cells. (**A**) The binding ability of peptides FITC-CM 7, FITC-CM 14, or FITC alone and MKN-45 cells expressing c-Met was detected by flow cytometry after incubation with 1 μM FITC-CM 7, FITC-CM 14, or FITC, respectively. (**B**) The binding ability of peptides FITC-CM 7, FITC-CM 14, or FITC alone and c-Met-negative-cell MKN-28 was detected by flow cytometry after incubation with 1 μM FITC-CM 7, FITC-CM 14, or FITC, respectively. (**C**) *In vitro* fluorescence imaging of c-Met under optimal conditions (1 μM, 1 h) showed high levels of fluorescent signals in the MKN-45 cells incubated with FITC-CM 7 compared with the MKN-45 cells incubated with FITC-CM 14 or FITC alone. (**D**) *In vitro* fluorescence imaging of c-Met under optimal conditions (1 μM, 1 h) did not show any fluorescent signals in MKN-28 cells incubated with FITC-CM 7, FITC-CM 14, or FITC alone. Scale bar = 10 μm.

Flow cytometry technology and a confocal microscope (LSM 800, Zeiss, Germany) were used to evaluate the binding ability between peptides FITC-CM 7, FITC-CM 14, or FITC and c-Met-positive or -negative cells in a physiological environment. 1,1′-Dioctadecyl-3,3′,3′-tetramethylindocarbocyanine perchlorate (DiI) is one of the most commonly used cell membrane fluorescence probes, presenting orange-red fluorescence. DiI is a type of lipophilic membrane dye that can spread laterally after entering the cell membrane to gradually stain the whole cell membrane (red), and Hoechst 33342 can stain the nucleus blue. The combination of FITC-CM 7, FITC-CM 14, or FITC with cells can make the cells green, and the merging of green, blue, and red layers can qualitatively achieve localization for the binding property. The expression of c-Met in MKN-45 and MKN-28 was detected by flow cytometry and Western blot, and the results are shown in Figure 3. c-Met was highly expressed in MKN-45 cells but not detected in MKN-28 cells. As shown in Figure 4A,B, 1 μM of FITC-CM 7 peptide exhibited significantly higher binding characteristics with MKN-45 than with MKN-28 cells, while the corresponding concentration of FITC-CM 14 or FITC alone hardly combined. Consistent with the flow cytometry results, except that 1 μM of FITC-CM 7 could bind better to MKN-45 cells and presented a fluorescence image as shown in Figure 4C,D, the corresponding concentration of FITC-CM 14 or FITC alone could not bind to MKN-45 or MKN-28 cells. The above results showed that FITC-CM 7 could specifically bind to c-Met-positive cells rather than nonspecifically bind to c-Met-negative cells. 

### 2.4. The c-Met-Targeting Peptide CM 7 Regulates c-Met-Driven Cell Proliferation 

**Figure 5:**
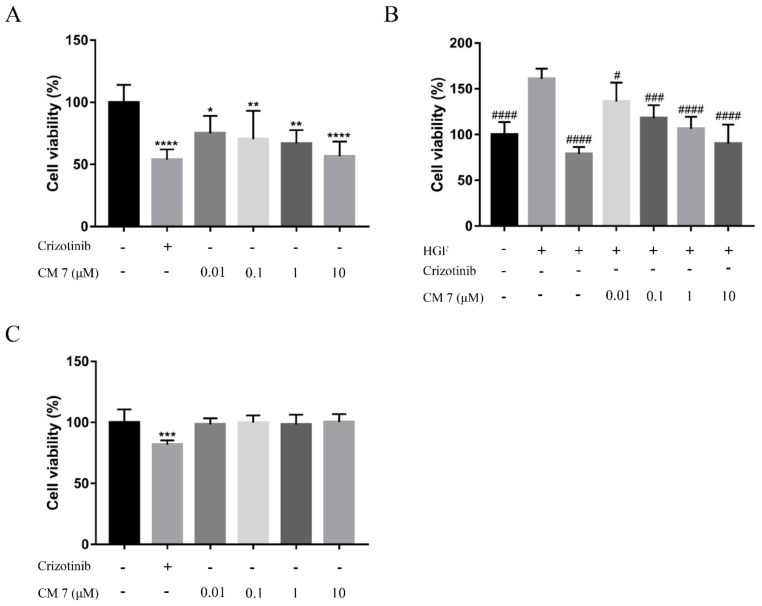
Cell proliferation assay: Inhibition of MKN-45, MKN-28, and U87 cell proliferation by the peptide CM 7. (**A**) MKN-45 cells were treated with various concentrations of CM 7, and cell viability was calculated after four days. (**B**) U87 cell proliferation was stimulated by 20 ng/mL HGF, cells were treated with various concentrations of CM 7, and cell viability was calculated after four days. (**C**) MKN-28 cells were treated with various concentrations of CM 7, and cell viability was calculated after four days. * *p* < 0.05, ** *p <* 0.01, **** *p <* 0.0001, indicate significant inhibition of MKN-45 or MKN-28 cell proliferation by CM 7 vs control; ^#^ *p <* 0.05, ^###^ *p <* 0.001, ^#####^ *p <* 0.0001, indicate significant inhibition of U87 cell proliferation by CM 7 vs HGF. Values are means of six different determinations; mean ± SD, one-way ANOVA with multiple comparisons.

MKN-45, MKN-28, and U87 cells were treated with the indicated CM 7 or HGF (20 ng/mL), and the proliferation of those three cells was counted and analyzed after four days. Crizotinib could significantly inhibit the proliferation of MKN-45, MKN-28, or U87 cells, and CM 7 could also inhibit the proliferation of MKN-45 or U87 cells to a certain extent with considerable dose dependence (Figure 5A,B), but it had no effect on MKN-28 cells (Figure 5C).

### 2.5. Peptide CM-7 Inhibits c-Met-Dependent Proliferation by Inducing S Arrest

**Figure 6:**
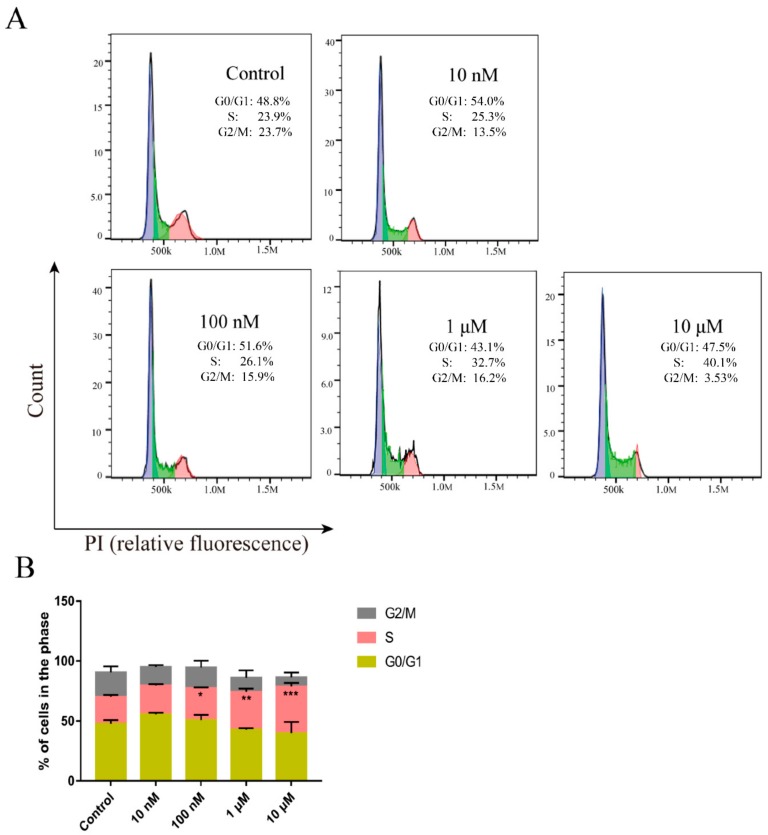
Effects of peptide CM 7 on cell cycle against MKN-45 cells. (**A**) MKN-45 cells were treated with the indicated concentrations of CM 7 (0, 0.01, 0.1, 1, and 10 μM) for 48 h; then, the cell cycle distribution was analyzed by flow cytometry. (**B**) Representative histograms for cell cycle distribution in MKN-45. Results are presented as mean ± SD (n = 3). * *p <* 0.05, ** *p <* 0.01, *** *p <* 0.001 vs control, one-way ANOVA with multiple comparisons.

The proliferation of tumor cells is affected by many factors, among which the regulation of cell cycle and apoptosis is the most common. Since peptide drugs are highly safe and tolerable [20], we mainly explored how peptide CM 7 affects the proliferation of MKN-45 gastric cancer cells from the perspective of cell cycle regulation. MKN-45 cells were significantly arrested in the S phase after treatment with peptide CM 7 compared with the control group (Figure 6A,B).

### 2.6. The c-Met-Targeting Peptide CM 7 Regulates c-Met-Dependent Cell Invasion, Migration, and Scattering

**Figure 7:**
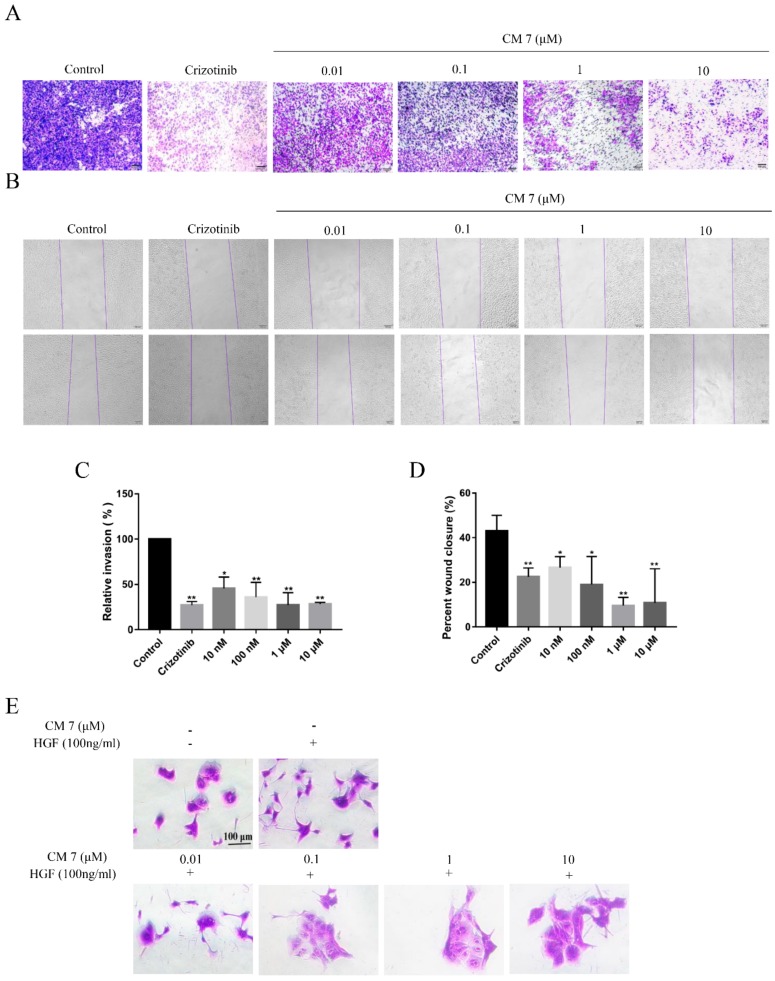
CM 7 suppresses c-Met-mediated cell invasion, migration, and scattering. (**A**) The invasion ability of MKN-45 cells was weakened by CM 7. (**B**) The migratory ability of MKN-45 cells was impaired by CM 7; the top is 0 h, and the bottom is 48 h. (**C**,**D**) Representative histograms of relative cell invasion or migration of MKN-45. Results are presented as mean ± SD (n = 3). * *p <* 0.05, ** *p <* 0.01 vs control, one-way ANOVA with multiple comparisons. (**E**) MDCK cell scattering induced by HGF was dose-dependently inhibited by CM 7. Representative images from three separate experiments are shown. In all of the pictures shown in this figure, scale bars = 100 μm.

It has been reported that the c-Met-mediated signaling pathway plays an important role in the metastasis of malignant tumors [21], so we evaluated the invasion and migration of MKN-45 c-Met-positive cells by CM 7 and Crizotinib. We found that CM 7 could significantly inhibit cell invasion (Figure 7A,C) and cell migration (Figure 7B,D) in a dose-dependent manner.

Activation of the HGF/c-Met signaling pathway can significantly promote cell scattering, which is the basis of tumor metastasis [22,23]. According to the studies [24,25], Madin–Darby canine kidney (MDCK) cells grow in clusters, but they can produce pseudopodia and convert into migratory fibroblasts after HGF stimulation. Thus, MDCK cells were used to assess the effect of CM 7 on cell-scattering behavior. As shown in Figure 7E, CM 7 could remarkably inhibit the HGF-induced cell scattering of MDCK cells in a dose-dependent manner, and the growth of cell pseudopodia was completely inhibited at the concentration of 1 μM. 

### 2.7. Peptide CM-7 Potently Inhibits c-Met Activation and Signaling in Cancer Cells

**Figure 8:**
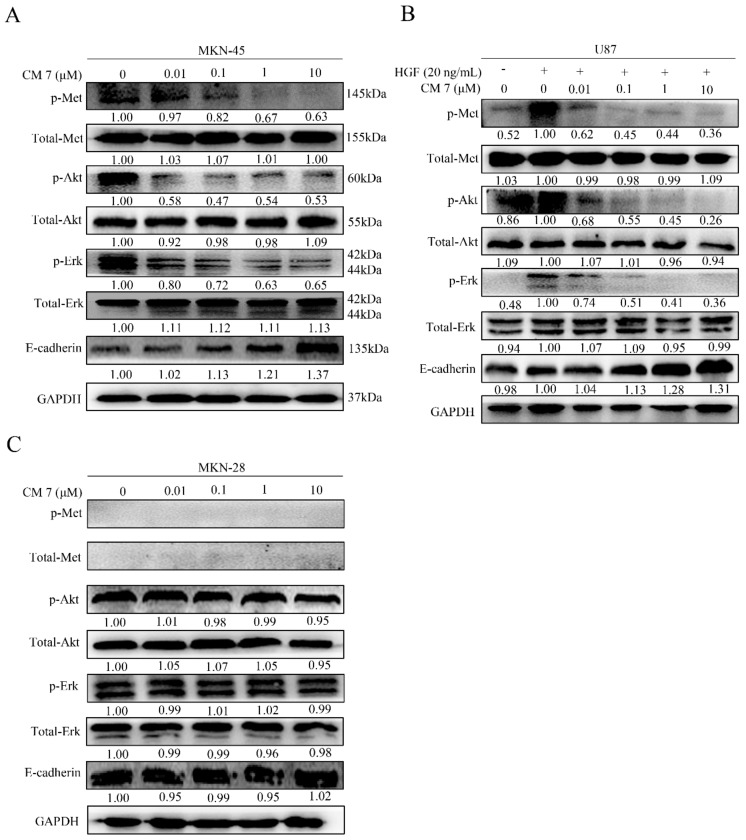
CM 7 inhibits c-Met phosphorylation and its downstream signaling molecules in different cells. (**A**) The phosphorylation of c-Met and downstream effector molecules Akt and Erk was inhibited by CM 7 in MKN-45 cells. MKN-45 cells were treated with the indicated concentrations of peptide CM 7 for 24 h, and the lysate was subjected to Western blot. (**B**) CM 7 suppresses HGF-induced c-Met phosphorylation and its downstream signaling molecules in U87 cells. U87 cells were treated with the indicated dose of peptide CM 7 for 24 h, stimulated without or with HGF for 15 min, then Western blot was performed to detect the proteins, as shown in the picture. (**C**) CM 7 had no effect on c-Met and its downstream signaling molecules in MKN-28 cells.

To further investigate how CM 7 affects cellular activity, we examined the phosphorylation of c-Met and its downstream associated signals after treatment of MKN-45, MKN-28, and U87 cells with CM 7. In these three types of cells, MKN-45 harbored typical *MET* amplification and c-Met was highly expressed, MKN-28 were the c-Met-negative cells, and U87 cells responded well to HGF stimulation. As shown in Figure 8, the results suggested that CM 7 could effectively inhibit the phosphorylation of c-Met and its downstream related molecules Akt and Erk in a dose-dependent manner in MKN-45 and U87 cells (Figure 8A,B), but not MKN-28 cells (Figure 8C). In addition, CM 7 could increase the expression of E-cadherin in MKN-45 and U87 cells but had no effect on MKN-28 cells (Figure 8A–C).

### 2.8. Peptide CM7 Inhibits c-Met-Mediated Tumor Growth In Vivo

**Figure 9:**
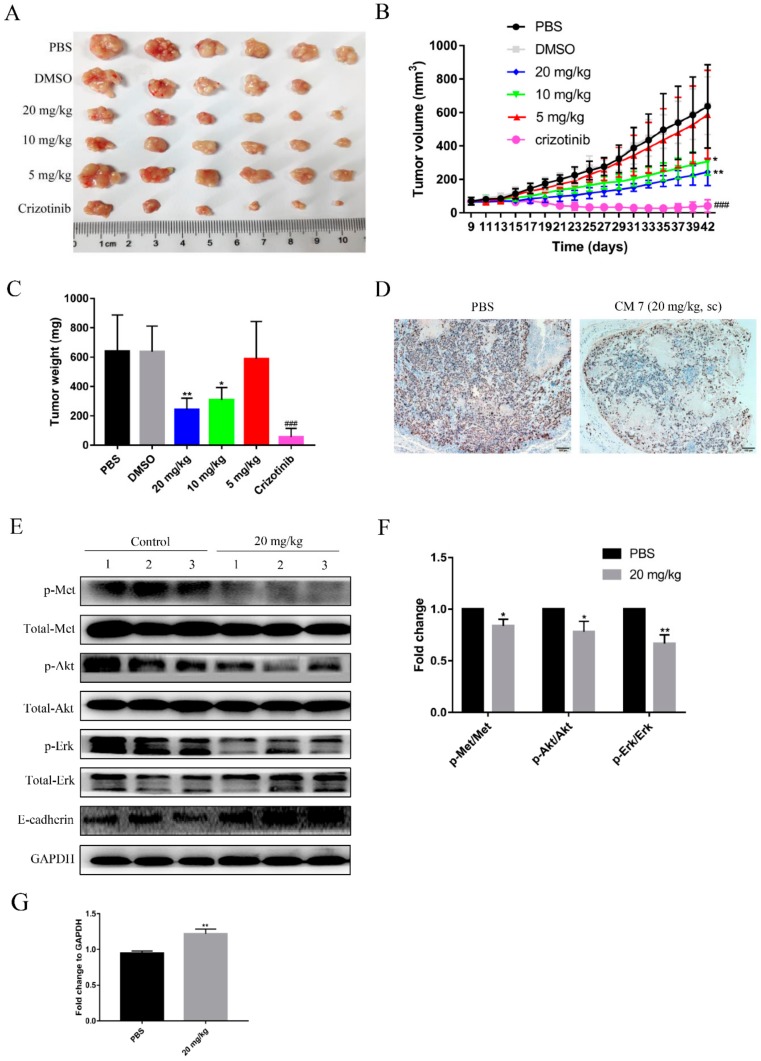
Inhibition of MKN-45 tumor growth by various treatments *in vivo*. Nude mice with tumor xenografts established by subcutaneous injection of MKN-45 cells were treated daily for four weeks with vehicle (0.2% DMSO) or Crizotinib (50 mg/kg) by gavage, and the tested peptide or vehicle (PBS) were subcutaneously injected twice a day for four weeks. (**A**) Images of the tumors of each group at the end of treatments. (**B**) The mean tumor volume of each group during the experimental period. (**C**) Tumor wet weights of each group were measured after the animals were sacrificed. Data are shown as mean ± SD obtained from each group (n = 5–6). * *p* < 0.05 vs vehicle (PBS) group, ** *p* < 0.01 vs vehicle (PBS) group, ### *p* < 0.001 vs vehicle (DMSO) group, unpaired *t*-test. (**D**) IHC evaluation of ki67 expression for the vehicle (PBS) and 20 mg/kg groups. (**E**) Inhibition of c-Met phosphorylation and downstream signaling molecules in MKN-45 xenografts by CM 7. (**F**,**G**) Quantitative statistics for the fold change of corresponding proteins in Figure 8E. Results are presented as mean ± SD (n = 3). * *p <* 0.05, ** *p <* 0.01 vs control, unpaired *t*-test.

The *in vivo* activities of peptide CM 7 were evaluated in a xenograft mouse model. MKN-45 human gastric cancer cells were subcutaneously injected into mice, then the mice were assigned randomly to six group (Table S1). The peptide CM 7 was subcutaneously injected twice a day for 28 days. Accordingly, Crizotinib was administered to tumor-bearing mice by gavage daily for 28 days. Tumor volume and weight were evaluated in this study, as shown in Figure 9A–C. The peptide CM 7, at the concentration of 20 mg/kg, significantly inhibited tumor growth compared with the vehicle control group (Phosphate buffer saline group, PBS group), but its inhibitory effect was weaker than the positive control group (Crizotinib). At the termination of the study, intratumoral ki67 was detected by immunohistochemistry analysis, and the expression of ki67 was deceased in the group that was administered at 20 mg/kg of peptide CM 7 (Figure 9D). Consistent with the results of *in vitro* experiments, phosphorylation of c-Met and its downstream related molecules Akt or Erk was also markedly inhibited intratumorally (Figure 9E,F). Further, the expression of E-cadherin was significantly increased (Figure 9G). In addition, no significant weight loss was observed in the mice in each peptide group, indicating that CM 7 was well tolerated and slightly or nontoxic (Figure S3).

## 3. Discussion

Currently, most of the marketed c-Met-targeted drugs are small-molecule drugs that are relatively highly toxic, have high production costs, and have a considerable number of side effects, which is not conducive to widespread clinical application. Therefore, the development of peptide drugs with relatively low toxicity and few side effects is of great research value. In addition, in terms of peptide drugs, they also have good drug resistance, low immunogenicity, and low production costs, so this study is also highly valuable in the field of practical application.

We designed peptides targeting the Sema or IPT domains of c-Met and detected the binding capacity using flow cytometry technology and laser confocal microscope imaging in living cells. The results of this study indicated that the peptide CM 7 could bind to c-Met protein at a high affinity, and a significant finding was that peptide CM 7 could regulate the functions of related cells, such as cell proliferation, invasion, migration, and scattering in a ligand-dependent or -independent way. As reported, the Sema domain of c-Met is necessary for HGF binding and receptor dimerization [26]. Based on our studies, we speculated that the inhibition of CM 7 may be due to the combination of CM 7 and the Sema domain affecting the dimerization of c-Met, or the interaction of CM 7 with c-Met, leading to the change of the conformation of the c-Met protein and thus regulating the phosphorylation of the receptor itself [26]. Further studies will be required to determine the precise role of CM 7 and its interaction with c-Met.

Unlike traditional cytotoxic drugs, targeted drugs target key molecules in cell signaling pathways that regulate the cell cycle or cell survival. According to the study [27], targeted drugs always need to be continuously administered multiple times within a reasonable dosage range in order to achieve sustained inhibition of the target, so as to achieve lasting and enhanced efficacy *in vivo*. However, considering the follow-up study, we thought that the effective dose of CM 7 was too high, which may be due to its short half-life in animals. As mentioned in literature reviews, PEGylation is one of the most successful strategies to improve drug properties, such as prolonging half-life, improving their pharmacokinetics and pharmacodynamics, and so on [28,29]. The stapling technique is also an important, and the most widely adopted, strategy to enhance stability or biological activity by introducing α helices into a peptide [30,31]. Thus, PEGylation of CM 7 or the formation of a staple peptide, through the introduction of α helices, provides a direction for future research and modification. In addition, nonstandard peptides are characterized by their structural and functional diversity, higher affinity, and physiological stability. Therefore, they can be used as strategies for further structural and functional optimization, such as the application of D-amino acids and L-amino acids with noncanonical side chains, cyclic structures, and even macrocyclic structures [32,33].

## 4. Materials and Methods 

### 4.1. Design and Screening of Peptides Targeting c-Met 

MOE software was used to analyze the interaction between c-Met and the HGF β chain (PDB ID:1SHY), and the structure of c-Met was modeled using I-TASSER [34,35,36]. Further, the interaction between c-Met and the HGF α chain (PDB ID:3HMS) was analyzed using the above-described method. We designed a virtual peptide library that can interact with c-Met based on the interaction between HGF and c-Met. These peptides were designed based on the corresponding HGF interacting fragment, and neutral or hydrophilic amino acids, such as Ile and Asn, were used to replace or insert into appropriate positions to improve their affinity with the receptor. Then, the MOE 2009 (Chemical Competing Group, Quebec, Canada) software was used for docking, and the scoring functions were Triangle Matcher and Forcefield.

### 4.2. Cell Culture

Human gastric cancer cell line MKN-45 cells were from the Chinese Academy of Medical Sciences, Beijing, China. Human gastric cancer cell line MKN-28 cells, the human glioma cell line U87 cells, and MDCK cells were obtained from the American Type Culture Collection (ATCC). The MKN-45 and MKN-28 cells were grown under a humidified atmosphere of 5% CO_2_ at 37 °C in RPMI 1640 medium (Biological Industries, Israel), supplemented with 10% fetal bovine serum (FBS, Biological Industries, Israel), 2 mmol/L l-glutamine (Gibco, Life Technologies, Grand Island, NY, USA), 100 U/mL penicillin, and 100 μg/mL streptomycin (Invitrogen, Carlsbad, CA, USA). MDCK and U87 cells were cultured under a humidified atmosphere of 5% CO_2_ at 37 °C in Dulbecco’s modified Eagle’s medium (DMEM) (Gibco, Life Technologies, Grand Island, NY, USA), supplemented with 10% FBS, 2 mmol/L l-glutamine (Gibco, Life Technologies, Grand Island, NY, USA), 100 U/mL penicillin, and 100 μg/mL streptomycin (Invitrogen, Carlsbad, CA, USA).

### 4.3. Peptide Preparation

The solid-phase peptide synthesis (SPPS) was performed to synthesize the peptide using the Focus XC system (AAPPTec, Louisville, KY, USA). During the synthesis process, 1-hydroxbenzotriazole (HOBt) and *N*,*N*′-diisopropylcarbodiimide (DIC) served as the peptide coupling reagent, and piperidine (PIP) was applied as the deprotection reagent. After synthesis, 90% trifluoracetic acid (TFA) was used for cutting and ether was used for precipitation. The synthesized crude peptide was purified by HPLC, and the purity and molecular mass were determined by HPLC or ESI-MS. In our study, FITC-CM 7 and FITC-CM 14 were the fluorescent-peptide-linked FITC with CM 7 and CM 14, respectively, via an Acp linker, and the above-described purification and identification methods were used.

### 4.4. Flow Cytometry Analysis

A flow cytometry assay was used to evaluate the conjugation and affinity of the newly designed peptides CM 7, CM 14, or FITC alone to c-Met-positive MKN-45 cells and c-Met-negative MKN-28 cells [37]. Briefly, MKN-45 and MKN-28 cells were detached by 0.25% trypsin and the final cell concentration was adjusted to 3 × 10^6^ cells/mL. The cell suspension (100 μL) was co-incubated with FITC-CM 7, FITC-CM 14, or FITC alone at the final concentration of 1 μM under humid conditions, 5% CO_2_, and room temperature for 1 h in a dark environment. After that, cells were collected, washed, and investigated by a MACS Quant flow cytometer (Miltenyi Biotec GmbH, Bergisch Gladbach, Germany). FloJo software version 7.6 (FlowJo LLC, Ashland, USA) was employed to analyze the data.

### 4.5. Confocal Microscope Imaging Analysis

MKN-45 and MKN-28 cells were plated separately in a glass-bottom cell culture dish (NEST) with serum-free media overnight. The next day, cells were fixed with 4% paraformaldehyde for 20 min at room temperature. After that, cells were washed with PBS and blocked with 1% bovine serum albumin (BSA, Fcmacs Biotech, Nanjing, China) for 30 min at room temperature; then, the cells were incubated with a final concentration of 1 μM of peptide FITC-CM 7, FITC-CM 14, and FITC (Solarbio, Shanghai, china) alone for 1 h at room temperature away from the light. Following that, cells were washed with PBS twice and stained with 8 μM of DiI for 10 min at room temperature. Subsequently, cells were washed with PBS twice and stained with Hoechst 33342 (Wanleibio, Shenyang, China) for 20 min at room temperature. Finally, cells were washed with PBS three times and visualized under a confocal microscope (LSM 800, Zeiss, Germany) with an oil-immersion objective lens (×63, Plan-Apochromatlan, Zeiss, Germany).

### 4.6. Cell Proliferation Assays 

As described in the literature [38], MKN-45, MKN-28, and U87 cells were seeded into 48-well culture plates (6500 cells/well) and cultured overnight. The next day, the cells were washed with PBS gently, and fresh serum-free medium with or without recombinant human HGF (20 ng/mL, PeproTech, Rocky Hill, NJ, USA) and CM 7 was added to the corresponding wells. After 4 days, the cells were trypsinized and counted by a cell counter. Crizotinib (Dumabio, Shanghai, China), an antitumor drug that targets the RTK c-Met, anaplastic lymphoma kinase (ALK), and repressor of silencing 1 (ROS1), was chosen (40 nM) to be tested as a positive control [39,40].

### 4.7. Cell Invasion and Wound Healing Assays

For the invasion assay [41,42], a chamber was placed into a 24-well culture plate with 600 μL RPMI 1640 medium containing 10% FBS. MKN-45 cells were resuspended in serum-free medium and cultured on the upper compartment of the Transwell chamber (1.0 × 10^5^ cells/well; pore size, 8 μm; Merck Millipore, Germany) coated with Matrigel. CM 7 and Crizotinib were added to the top side of the membrane at the indicated concentrations. After 48 h, the noninvaded cells were removed, and the invaded cells were fixed with ethanol and stained with crystal violet solution (0.1%) for 15 min at room temperature. The experiment was performed in triplicate, and images were obtained using an Olympus IX53 microscope (Olympus, Japan). 

For the wound healing assay [43], MKN-45 cells (4 × 10^4^ cells/well) were seeded in a 48-well culture plate. When the cells were almost completely fused, a sterile 200 μL pipette tip was used to generate a straight wound line across the monolayer cell. Then, the cells were washed with PBS and incubated in RPMI 1640 medium containing 1% FBS with CM 7 or Crizotinib at the indicated concentration for 48 h. The experiment was performed in triplicate, and the cell migration toward the gap was captured using the microscope. 

### 4.8. Cell Scatter Assay

MDCK cells were detached from the tissue culture plate and seeded in 96-well plates at the final concentration of 1.5 × 10^3^ cells/well overnight. Different concentrations of CM 7 without or with HGF (100 ng/mL) were added to the preset wells. After incubating for 24 h at 37 °C and 5% CO_2_, 4% paraformaldehyde was used to fix the cells, which were then stained with 0.2% crystal violet. The plates were washed with clean water. When dried, an Olympus IX53 microscope was used to capture the images.

### 4.9. Cell Cycle Distribution Assay

According to the cell cycle detection kit (Keygen) instructions, MKN-45 cells were collected and seeded in a six-well culture plate at the concentration 2 × 10^6^/mL overnight. Then, the cells were washed with PBS and treated with peptide CM 7 at the indicated concentration for 48 h. The experiment was performed in triplicate. A MACS Quant flow cytometer (Miltenyi Biotec GmbH, Bergisch Gladbach, Germany) was used to detect the cell cycle profile, and FlowJo software version 7.6 (FlowJo LLC, Ashland, USA) was used to analyze the data.

### 4.10. Western Blot Analysis

Western blot analysis was performed using an extraction buffer as described [44]. MKN-45, MKN-28 and U87 cells were cultured in serum-free medium overnight, then treated with the indicated dose of peptide CM 7 for 24 h at 37 °C after stimulated without or with HGF for 15 min. Next, total cell lysates were separated by 12% SDS-polyacrylamide gel (SDS-PAGE) and transferred to polyvinylidene difluoride (PVDF) membranes. The PVDF membranes were probed with following primary antibodies: anti-phospho-Met (Tyr1234/1235, (Cell Signaling Technology, Beverly, MA, USA), anti-Met (Affinity, USA), anti-phospho-AKT (Ser473, (Affinity, USA)), anti-AKT (Affinity, USA), anti-phospho-Erk1/2 (Thr202/Tyr204, (Affinity, USA)), anti-Erk1/2 (Affinity, USA), anti-E-cadherin (WanleiBio, Shengyang, China), and anti-GAPDH (Multi Sciences, Hangzhou, China). Then, horseradish-peroxidase-conjugated anti-rabbit IgG was used as a second antibody. The immunoreactive proteins were exposed using an enhanced chemiluminescence detection reagent (WanleiBio, Shengyang, China). After sacrificing the mice and isolating the tumor tissue, Western blot was performed as previously described. 

### 4.11. In Vivo Antitumor Activity Assay

Four-to-five-week-old female Balb/c nude mice, housed in specific pathogen-free conditions, were used to evaluate antitumor activity *in vivo*. All animal studies were performed according to the institutional ethical guidelines for animal care.

MKN-45 cells at a density of 5 × 10^6^ in 200 μL were injected subcutaneously into the flank of the mice. CM 7 (5, 10, or 20 mg/kg) was injected subcutaneously for each group twice a day for four weeks. As a positive control, Crizotinib (50 mg/kg) was given to tumor-bearing mice by gavage once a day for a total of 28 days. The tumor volume was measured with calipers in two dimensions: tumor volume = 1/2 × a × b^2^, where a is the length of the tumor, and b is the width of the tumor. All of the mice were sacrificed on day 42, and the tumor tissue was isolated and weighed. 

### 4.12. Immunohistochemistry Assay

After being fixed with 10% formalin solution for 24 h, the tumor tissues were transferred to 70% ethanol. The tumor specimens were embedded in paraffin, sliced with a slicer, and backed on microscope slides. The slides were incubated with primary antibody (ki67 antibody purchased from Servicebio, Wuhan, China) and secondary antibody sequentially. Finally, the slides were exposed to DAB (Servicebio, Wuhan, China) for visualization. Images were captured by an Olympus IX53 microscope. 

## 5. Conclusions

The findings of this study show that the novel peptide CM 7 could specifically bind to c-Met-positive cells and inhibit c-Met-mediated cell proliferation, invasion, migration, and scattering by arresting the cell cycle in the S phase or blocking the phosphorylation of c-Met and its downstream signaling molecules. As expected, *in vivo* results also show that CM 7 could inhibit tumor progression and development to some extent. Therefore, CM 7 may be a promising candidate compound with research and application value that can provide a basis for accurate c-Met-mediated clinical treatment of tumors.

## Figures and Tables

**Table 1:** Virtual screening of c-Met and designed peptides.

Number	Sequences	Scoring	
		Sema	IPT
CM 1	ADQCANRCT	−7.01	−7.48
CM 2	VPGRGC	−5.94	−6.77
CM 3	DQIANRC	−7	−7.19
CM 4	DQCANR	−7.02	−7.4
CM 5	DQIANR	−6.69	−7.86
CM 6	DQIANN	−5.98	−6.84
CM 7	DQIIANN	−7.07	−7.23
CM 8	VPGRGD	−6.54	−6.81
CM 9	VPGRGS	−6.28	−6.68
CM 10	VPGNGS	−6.42	−6.58
CM 11	VNGRGS	−6.26	−7.04
CM 12	VQGRGS	−6.15	−6.92
CM 13	VQGRGC	−6.16	−7.64
CM 14	QTRIYWQKE	−5.81	−6.14

## Supplementary Materials

Figure S1: The chemical structure, molecular mass, and purity of peptide FITC-CM 7. (A) Chemical structure of peptide FITC-CM 7. The peptide and FITC were coupled with an Acp linker. (B) Electrospray ionization mass spectrometry (ESI-MS) analysis showed that the molecular mass of peptide FITC-CM 7 was 1289. (C) The purity of peptide FITC-CM 7 was determined by high-performance liquid chromatography (HPLC) to be 97.3%. Figure S2: The chemical structure, molecular mass, and purity of peptide FITC-CM 14. (A) Chemical structure of peptide FITC-CM 14. The peptide and FITC were coupled with an Acp linker. (B) ESI-MS analysis showed that the molecular mass of peptide FITC-CM 14 was 1754. (C) The purity of peptide FITC-CM 7 was determined by HPLC to be 96.7%. Figure S3: The mean body weight of tumor-bearing mice treated daily for four weeks with vehicle (0.2% DMSO) or Crizotinib (50 mg/kg) by gavage, and the tested peptide CM 7 or vehicle (PBS) that was subcutaneously injected twice a day for four weeks was measured every other day during the study. Table S1: Treatment groups.

## References

[1] Bottaro D.P., Rubin J.S., Faletto D.L., Chan A.M.L., Kmiecik T.E., Woude G.F.V., Aaronson S.A. (1991). Identification of the hepatocyte growth factor receptor as the c-met proto-oncogene product. Science.

[2] Giordano S., Ponzetto C., Direnzo M.F., Cooper C.S., Comoglio P.M. (1989). Tyrosine kinase receptor indistinguishable from the c-met protein. Nature.

[3] Tempest P.R., Stratton M.R., Cooper C.S. (1988). Structure of the met protein and variation of met protein kinase activity among human tumour cell lines. Br. J. Cancer.

[4] Stamos J., Lazarus R.A., Yao X., Kirchhofer D., Wiesmann C. (2004). Crystal structure of the HGF b-chain in complex with the Sema domain of the Met receptor. EMBO J..

[5] Vigna E., Chiriaco C., Cignetto S., Fontani L., Basilico C., Petronzelli F., Comoglio P.M. (2015). Inhibition of ligand-independent constitutive activation of the Met oncogenic receptor by the engineered chemically-modified antibody DN30. Mol. Oncol..

[6] Basilico C., Arnesano A., Galluzzo M., Comoglio P.M., Michieli P. (2008). A high affinity hepatocyte growth factor-binding site in the immunoglobulin-like region of Met. J. Biol. Chem..

[7] Miller K.D., Nogueira L., Mariotto A.B., Rowland J.H., Yabroff K.R., Alfano C.M., Jemal A., Kramer J.L., Siegel R.L. (2019). Cancer treatment and survivorship statistics, 2019. CA Cancer J. Clin..

[8] Ma W.W., Adjei A.A. (2009). Novel agents on the horizon for cancer therapy. CA Cancer J. Clin..

[9] Trusolino L., Comoglio P.M. (2002). Scatter-factor and semaphorin receptors: cell signalling for invasive growth. Nat. Rev. Cancer.

[10] Suzuki Y., Sakai K., Ueki J., Xu Q., Nakamura T., Shimada H., Nakamura T., Matsumoto K. (2010). Inhibition of Met/HGF receptor and angiogenesis by NK4 leads to suppression of tumor growth and migration in malignant pleural mesothelioma. Int. J. Cancer.

[11] Birchmeier C., Birchmeier W., Gherardi E., Vande Woude G.F. (2003). Met, metastasis, motility and more. Nat. Rev. Mol. Cell Biol..

[12] Trusolino L., Bertotti A., Comoglio P.M. (2010). MET signalling: Principles and functions in development, organ regeneration and cancer. Nat. Rev. Mol. Cell Biol..

[13] Schmidt C., Bladt F., Goedecke S., Brinkmann V., Zschiesche W., Sharpe M., Gherardi E., Birchmeier C. (1995). Scatter factor/hepatocyte growth factor is essential for liver development. Nature.

[14] Borowiak M., Garratt A.N., Wüstefeld T., Strehle M., Trautwein C., Birchmeier C. (2004). Met provides essential signals for liver regeneration. Proc. Natl. Acad. Sci. USA.

[15] Gavine P.R., Ren Y., Han L., Lv J., Fan S., Zhang W., Xu W., Liu Y.J., Zhang T., Fu H. (2015). Volitinib, a potent and highly selective c-Met inhibitor, effectively blocks c-Met signaling and growth in c-MET amplified gastric cancer patient-derived tumor xenograft models. Mol. Oncol.

[16] Burggraaf J., Kamerling I.M.C., Gordon P.B., Schrier L., de Kam M.L., Kales A.J., Bendiksen R., Indrevoll B., Bjerke R.M., Moestue S.A. (2015). Detection of colorectal polyps in humans using an intravenously administered fluorescent peptide targeted against c-Met. Nat. Med..

[17] Zhang H., Feng Q., Chen W.D., Wang Y.D. (2018). HGF/c-MET: A Promising Therapeutic Target in the Digestive System Cancers. Int. J. Mol. Sci..

[18] Wu Y., Fan Q., Zeng F., Zhu J., Chen J., Fan D., Li X., Duan W., Guo Q., Cao Z. (2018). Peptide-Functionalized Nanoinhibitor Restrains Brain Tumor Growth by Abrogating Mesenchymal-Epithelial Transition Factor (MET) Signaling. Nano. Lett..

[19] Zhao M., Wang Y., Liu Y., Zhang W., Liu Y., Yang X., Cao Y., Wang S. (2019). C7 peptide inhibits hepatocellular carcinoma metastasis by targeting the HGF/c-Met signaling pathway. Cancer Biol. Ther..

[20] Pockley A.G., Lindsay J.O., Foulds G.A., Rutella S., Gribben J.G., Alexander T., Snowden J.A. (2018). Immune Reconstitution After Autologous Hematopoietic Stem Cell Transplantation in Crohn’s Disease: Current Status and Future Directions. A Review on Behalf of the EBMT Autoimmune Diseases Working Party and the Autologous Stem Cell Transplantation In Refractory CD-Low Intensity Therapy Evaluation Study Investigators. Front. Immunol..

[21] Zhang Y., Su Y., Volpert O.V., Woude G.F.V. (2003). Hepatocyte growth factor/scatter factor mediates angiogenesis through positive VEGF and negative thrombospondin 1 regulation. Proc. Natl. Acad. Sci. USA.

[22] Nakamura T., Nawa K., Ichihara A. (1984). Partial purification and characterization of hepatocyte growth factor from serum of hepatectomized rats. Biochem. Biophys. Res. Commun..

[23] Al-U’datt D.G.F., Al-Husein B.A.A., Qasaimeh G.R. (2017). A mini-review of c-Met as a potential therapeutic target in melanoma. Biomed. Pharmacother..

[24] Thiery J.P. (2002). Epithelial-mesenchymal transitions in tumour progression. Nat. Rev. Cancer.

[25] Lai J.K., Wu H.C., Shen Y.C., Hsieh H.Y., Yang S.Y., Chang C.C. (2012). Kruppel-like factor 4 is involved in cell scattering induced by hepatocyte growth factor. J. Cell Sci..

[26] Kong-Beltran M., Stamos J., Wickramasinghe D. (2004). The Sema domain of Met is necessary for receptor dimerization and activation. Cancer Cell.

[27] Kwak E.L., Bang Y.J., Camidge D.R., Shaw A.T., Solomon B., Maki R.G., Ou S.-H., Dezube B.J., Jänne P.A., Costa D.B. (2010). Anaplastic lymphoma kinase inhibition in non-small-cell lung cancer. N. Engl. J. Med..

[28] Turecek P.L., Bossard M.J., Schoetens F., Ivens I.A. (2016). PEGylation of Biopharmaceuticals: A Review of Chemistry and Nonclinical Safety Information of Approved Drugs. J. Pharm. Sci..

[29] Milla P., Dosio F., Cattel L. (2012). PEGylation of proteins and liposomes: a powerful and flexible strategy to improve the drug delivery. Curr. Drug Metab..

[30] Tan Y.S., Lane D.P., Verma C.S. (2016). Stapled peptide design: principles and roles of computation. Drug Discov. Today.

[31] Lau Y.H., Wu Y., Rossmann M., Tan B.X., de Andrade P., Tan Y.S., Verma C., McKenzie G.J., Venkitaraman A.R., Hyvonen M. (2015). Double Strain-Promoted Macrocyclization for the Rapid Selection of Cell-Active Stapled Peptides. Angew. Chem. Int. Ed. Engl..

[32] Hwang D.W., Bahng N., Ito K., Ha S., Kim M.Y., Lee E., Suga H., Lee D.S. (2017). In vivo targeting of c-Met using a non-standard macrocyclic peptide in gastric carcinoma. Cancer Lett..

[33] Miao W., Sakai K., Imamura R., Ito K., Suga H., Sakuma T., Yamamoto T., Matsumoto K. (2018). MET Activation by a Macrocyclic Peptide Agonist that Couples to Biological Responses Differently from HGF in a Context-Dependent Manner. Int. J. Mol. Sci..

[34] Anderson D.M., Anderson K.M., Chang C.L., Makarewich C.A., Nelson B.R., McAnally J.R., Kasaragod P., Shelton J.M., Liou J., Bassel-Duby R. (2015). A micropeptide encoded by a putative long noncoding RNA regulates muscle performance. Cell.

[35] Zhang Y. (2008). I-TASSER server for protein 3D structure prediction. BMC Bioinform..

[36] Roy A., Kucukural A., Zhang Y. (2010). I-TASSER: A unified platform for automated protein structure and function prediction. Nat. Protoc..

[37] Li C., Zhang N., Zhou J., Ding C., Jin Y., Cui X., Pu K., Zhu Y. (2018). Peptide Blocking of PD-1/PD-L1 Interaction for Cancer Immunotherapy. Cancer Immunol. Res..

[38] Brockmann M.A., Papadimitriou A., Brandt M., Fillbrandt R., Westphal M., Lamszus K. (2003). Inhibition of Intracerebral Glioblastoma Growth by Local Treatment with the Scatter Factor/Hepatocyte Growth Factor-Antagonist NK4. Clin. Cancer. Res..

[39] Okamoto W., Okamoto I., Arao T., Kuwata K., Hatashita E., Yamaguchi H., Sakai K., Yanagihara K., Nishio K., Nakagawa K. (2012). Antitumor action of the MET tyrosine kinase inhibitor crizotinib (PF-02341066) in gastric cancer positive for MET amplification. Mol. Cancer. Ther..

[40] Heigener D.F., Reck M. (2018). Crizotinib. Recent Results Cancer Res..

[41] He C.X., Ai J., Xing W.Q., Chen Y., Zhang H.T., Huang M., Hu Y.H., Ding J., Geng M.Y. (2014). Yhhu3813 is a novel selective inhibitor of c-Met kinase that inhibits c-Met-dependent neoplastic phenotypes of human cancer cells. Acta Pharmacol. Sin..

[42] Justus C.R., Leffler N., Ruiz-Echevarria M., Yang L.V. (2014). In vitro cell migration and invasion assays. J. Vis. Exp..

[43] Li M., Han Y., Zhou H., Li X., Lin C., Zhang E., Chi X., Hu J., Xu H. (2018). Transmembrane protein 170B is a novel breast tumorigenesis suppressor gene that inhibits the Wnt/beta-catenin pathway. Cell Death Dis..

[44] Longati P., Bardelli A., Ponzetto C., Naldini L., Comoglio P.M. (1994). Tyrosines1234–1235 are critical for activation of the tyrosine kinase encoded by the MET proto-oncogene (HGF receptor). Oncogene.

