# Supplementary material for: Novel Peptide CM 7 Targeted c-Met with Antitumor Activity
Source: Molecules. 2020 Jan 21;25(3):451. doi: 10.3390/molecules25030451 (PMC7038139; doi:10.3390/molecules25030451)
Supplement: Supplementary file 1 [file molecules-25-00451-s001.pdf]

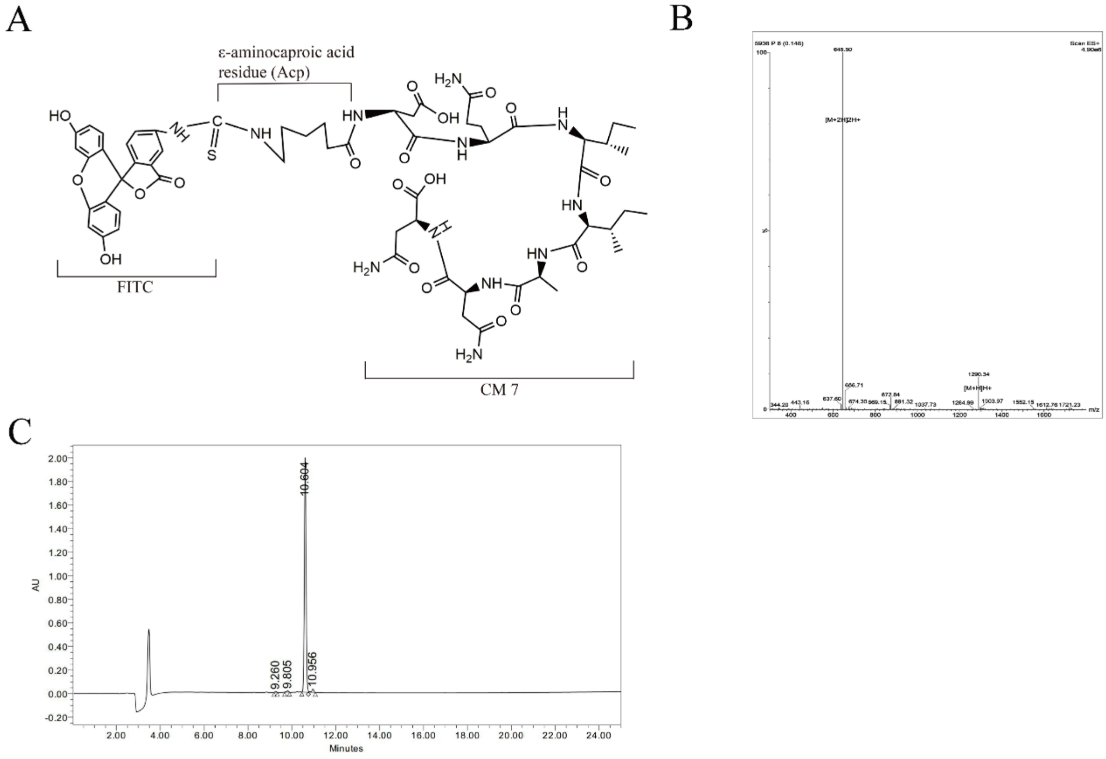

Figure S1. The chemical structure, molecular mass, and purity of peptide FITC-CM 7. (A) Chemical structure of peptide FITC-CM 7. The peptide and FITC were coupled with an Acp linker. (B) Electrospray ionization mass spectrometry (ESI-MS) analysis showed that the molecular mass of peptide FITC-CM 7 was 1289. (C) The purity of peptide FITC-CM 7 was determined by high-performance liquid chromatography (HPLC) to be 97.3%.

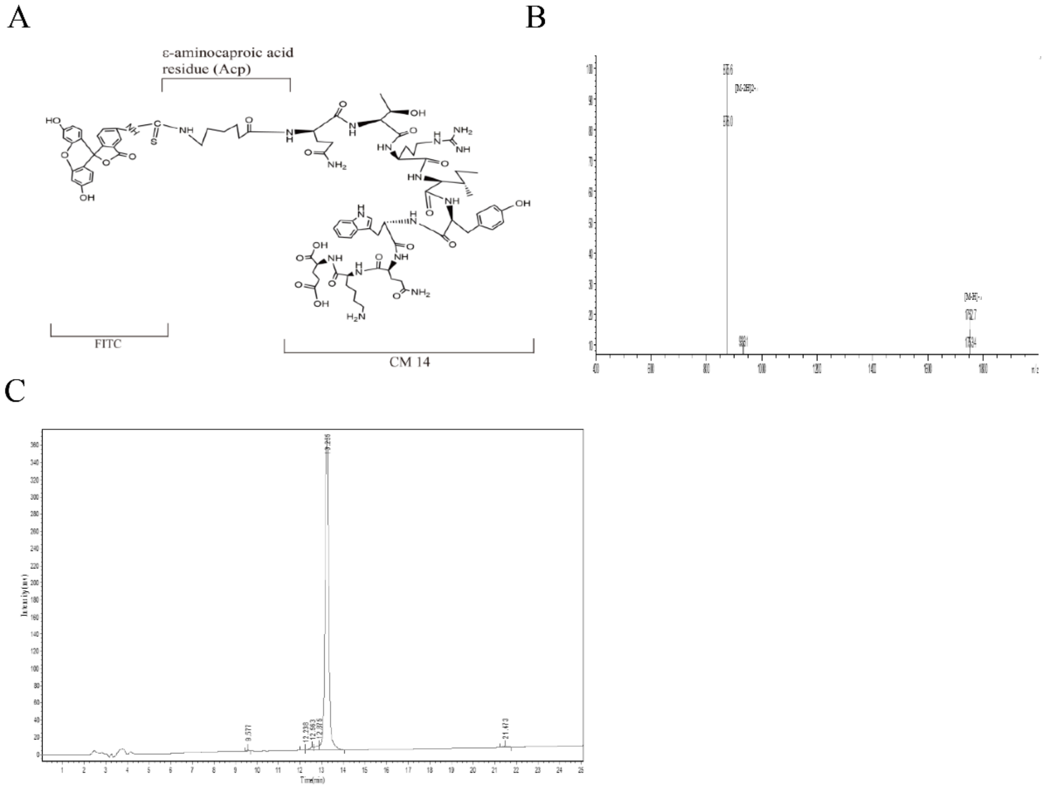

Figure S2. The chemical structure, molecular mass, and purity of peptide FITC-CM 14. (A) Chemical structure of peptide FITC-CM 14. The peptide and FITC were coupled with an Acp linker. (B) ESI-MS analysis showed that the molecular mass of peptide FITC-CM 14 was 1754. (C) The purity of peptide FITC-CM 7 was determined by HPLC to be 96.7%.

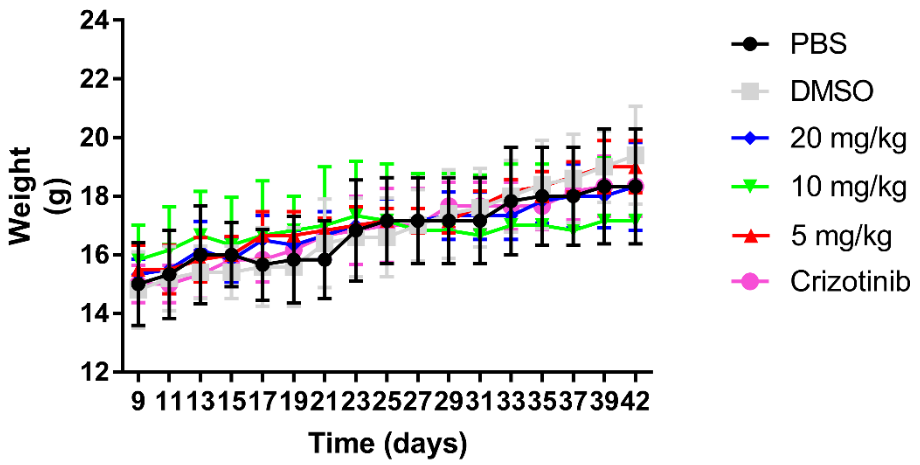

Figure S3: The mean body weight of tumor-bearing mice treated daily for four weeks with vehicle (0.2% DMSO) or crizotinib (50 mg/kg) by gavage, and the tested peptide CM 7 or vehicle (PBS) that was subcutaneously injected twice a day for four weeks was measured every other day during the study.

Table 1. Treatment groups.

Group	Treatment
PBS	Normal saline (NS), vehicle control (peptide CM 7), sc, Bid
DMSO	0.2% DMSO, vehicle control (Crizotinib), ig, qd
High dose group	20 mg/kg, sc, Bid
Middle dose group	10 mg/kg, sc, Bid
Low dose group	5 mg/kg, sc, Bid
Crizotinib	50 mg/kg, ig, qd
